# Environmental Consequences of Rapid Urbanization in Zhejiang Province, East China

**DOI:** 10.3390/ijerph110707045

**Published:** 2014-07-11

**Authors:** Xuchao Yang, Wenze Yue, Honghui Xu, Jingsheng Wu, Yue He

**Affiliations:** 1 Ocean College, Zhejiang University, Hangzhou 310058, China; E-Mail: yangxuchao@zju.edu.cn; 2 Zhejiang Institute of Meteorological Sciences, Hangzhou 310008, China; E-Mail: forsnow@126.com; 3 Department of Land Management, Zhejiang University, Hangzhou 310058, China; 4 Zhejiang Meteorological Information Center, Hangzhou 310017, China; E-Mail: wjs_hz@163.com; 5 Zhejiang Climate Center, Hangzhou 310017, China; E-Mail: heyue0925@163.com

**Keywords:** urbanization, urban heat island, extreme heat event, visibility, air pollution

## Abstract

Since reforms carried out in the late 1970s, China has experienced unprecedented rates of urban growth. Remote sensing data and surface observational data are used to investigate the urbanization process and related environmental consequences, focusing on extreme heat events and air pollution, in Zhejiang Province (ZJP, East China). Examination of satellite-measured nighttime light data indicates rapid urbanization in ZJP during the past decade, initially forming three urban clusters. With rapid urban sprawl, a significant Urban Heat Island (UHI) effect has emerged. During extreme heat events in summer, the UHI effect significantly exacerbates nocturnal heat stress in highly urbanized areas. Taking a long-term view, urbanization also causes additional hot days and hot degree days in urban areas. Urbanization also imposes a heavy burden on local and regional air quality in ZJP. Degraded visibility and an increase in haze days are observed at most meteorological stations, especially in the three urban clusters. The results show that urbanization has led to serious environmental problems in ZJP, not only on the city scale, but also on the regional scale. Maintaining a balance between the continuing process of urbanization and environmental sustainability is a major issue facing the local government.

## 1. Introduction

China has been undergoing a period of economic reform and expansion since the late 1970s. Concurrent with economic growth and industrialization is rapid and widespread urbanization. The demographics and physical landscapes of China are increasingly urban. From 1978 to 2011, China’s urbanization level rose from 18% to 51% [1]. While rapid urbanization has greatly accelerated economic and social development, it has also engendered numerous environmental problems, manifested in local climate alteration [2,3,4,5], carbon storage [6], increased air and water pollution [7,8,9], increased energy demands [10], a major reduction in natural vegetation production [11,12,13,14], and decreased ecosystem services [15]. Thus, identification and assessment of climatic and environmental impacts resulting from contemporary urbanization in China have become a top priority and have attracted increasing attention in recent years [8,16,17,18]. 

The urban heat island (UHI) effect is a well-documented phenomenon wherein a thermal anomaly exists in urban areas in comparison with proximate rural landscapes. Although a large effort has been devoted to the study of the UHI phenomenon, relatively little information is available in the literature about the impact of urbanization on extreme heat events. Air pollution is another environmental consequence of rapid urbanization that is associated with a wide range of adverse health outcomes. However, a common problem in conducting air pollution research in developing countries like China is the scarcity of quality long-term air monitoring data. Degraded visibility is the most direct side effect of high atmospheric particle concentrations. The resulting visual haziness is closely related to air quality. Visibility data are routinely collected at airports or meteorological stations, and are thus available for interpolation of missing pollutant measurements in developing countries [19]. It is worth noting that efforts to understand the environmental consequences of rapid urbanization have focused on the single-city scale, such as Shanghai [20,21,22], Beijing [23], Shenzhen [18,24], and Iskenderun [25], but little is known about to what extent the development of urban agglomeration influences the environment, such as climate and air quality, at the regional scale.

Zhejiang province (ZJP) is located on the east coast of China. As a representative of the eastern-coast developed provinces of China, ZJP has experienced rapid economic development and a dramatic growth in its urban population and urbanized areas since the late 1970s. To date, however, long-term and spatially explicit monitoring of the urbanization of ZJP, together with comprehensive studies of the environmental consequences, have not been conducted. The aim of this study is to explore the urban growth in ZJP and focus on the impacts of the rapid urbanization process on extreme heat events and air quality which are closely related to human health in cities. To this end, observational data from satellite measurements and ground station records were used. As a representative of rapid urbanization at the regional scale in China, the findings of the present research in ZJP provide significant evidence on the association between regional environmental deterioration and urbanization and have important implications for Chinese policy-makers in regional urban development planning.

## 2. Study Area and Data

### 2.1. Study Area

**Figure 1:**
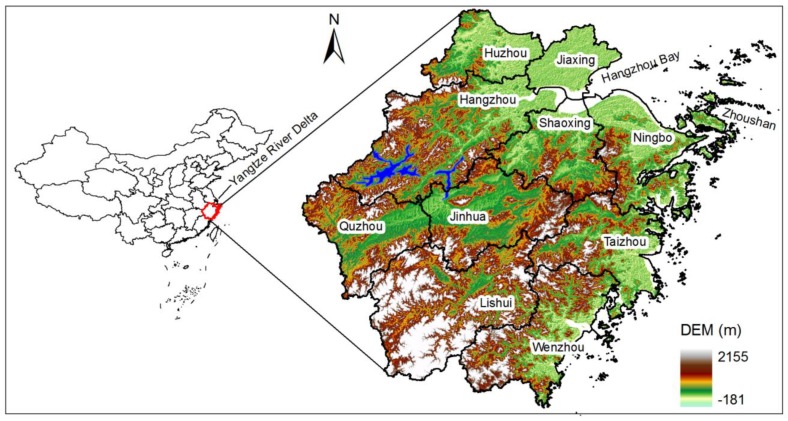
Location of the study area and its elevation.

Zhejiang province (ZJP) is located in the southern part of the Yangtze River Delta on the east coast of China (Figure 1). The province faces the East China Sea to the east and covers a total land area of 101,800 km^2^. ZJP has a complex terrain, with hills and mountains accounting for 70.4% of the total area. Plains and basins make up 23.2%, and the remaining 6.4% is water bodies (rivers and lakes). Under subtropical and monsoon conditions, ZJP has four distinct seasons, and plentiful sunshine. It has an average annual temperature ranging from 15.3 °C to 17.9 °C, 230–270 frost-free days and an average annual rainfall from 1000 to 1900 mm. 

**Figure 2:**
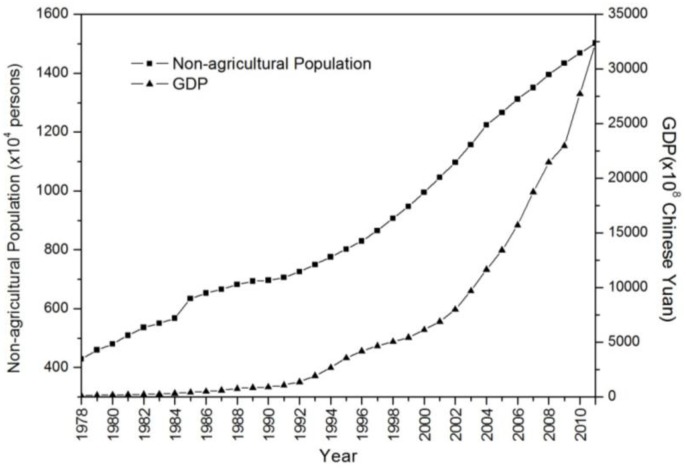
Changes in the non-agricultural population and the GDP from 1978–2011 for Zhejiang province.

The GDP of ZJP ranks fourth in the whole country although the total area ranks only 25th out of 34 provinces and municipalities. Entering the new millennium, there was a breakthrough in GDP increase (Figure 2). In addition, ZJP has also experienced a significant increase in urban population. The registered non-agricultural population has increased continuously from 4.29 million in 1978 to 15.02 million in 2011 [26]. 

### 2.2. Material and Methods

#### 2.2.1. DMSP/OLS Nighttime Light (NTL) Imagery

The National Oceanic and Atmospheric Administration’s National Geophysical Data Center (NGDC) generates annual stable light composites with 30 arc-second resolution. The stable light composite represents the average intensity of NTL in terms of digital number (DN) values ranging from 0 to 63. These stable NTL composites from 1992 to 2010 can be freely downloaded from the NGDC website [27]. The DMSP/OLS NTL data have been widely used to monitor the growth of built-up areas at regional scale [28,29,30,31] and high DN values in the NTL images generally indicate high fractional settlements. The stable NTL data were created using data from different sensors on different satellites. Therefore, the raw data cannot be used directly for temporal analyses due to the lack of inflight calibration [32,33,34]. Relative radiometric correction was implemented using the approach of Elvidge *et al.* [34]. The inter-calibrated NTL data for 1992 and 2010 used in this study were then clipped for ZJP. 

#### 2.2.2. Normalized Difference Vegetation Index (NDVI) Data

The moderate-resolution imaging spectroradiometer (MODIS) NDVI data (MOD13A3) in 2001 and 2011 at a resolution of 1 km were downloaded from the National Aeronautics and Space Administration (NASA) website. The MODIS Reprojection Tool (MRT) was used for image mosaic, format conversion and reprojection. A maximum value composite (MVC) method was used to generate annual maximum NDVI images for 2001 and 2011.

#### 2.2.3. Meteorological Data

Data on the observational meteorological elements, including temperature, relative humidity (RH), precipitation, visibility, and weather phenomena of 62 routine meteorological stations in ZJP were used in the study, covering the period 1960 to 2011. In addition, a dense network of automatic weather stations (AWSs) was deployed in 2005 across ZJP. The hourly temperature data from about 1000 AWSs in ZJP were used for the period of 2008–2012. All these data were acquired from the Zhejiang Meteorological Bureau and had undergone extensive automated quality control to eliminate many of the random errors found in the original data.

#### 2.2.4. Census Data

Census data of population, GDP, and the number of motor vehicles were obtained from the Zhejiang Provincial Bureau of Statistics [26].

#### 2.2.5. Air Quality Data

Monitoring data on nitrous dioxide (NO_2_) in Hangzhou City, the capital of ZJP, were provided by the Zhejiang Environmental Monitoring Center, a government agency responsible for air pollution data collection in ZJP.

#### 2.2.6. Classification of Stations

Based on DMSP/OLS NTL data, the non-agricultural population census data in 2010 and the method developed by Yang *et al.* [3], the 62 weather stations with long-term observational records were classified into 31 urban and 31 rural stations.

#### 2.2.7. Correction of Visibility

Surface visibility was recorded four times daily at each station; observation times were 02:00, 08:00, 14:00, and 20:00 (Beijing time). As observations at 02:00 and 20:00 were at night and at dusk, respectively, the selected reference objects are different from those of the daytime and cause the data discordance. Visibility at 08:00 was easily affected by radiation fog, as well as the surface inversion formed at night, which might impact the visibility at this time. Consequently, only visibility at 14:00 was used in the analysis to refiect annual visibility changes accurately. Moreover, only stations with continuous records after 1980 are presented within this paper, due to different methods of data collection before 1980. In order to reduce the effect of RH on visibility, the method from Rosenfeld *et al.* [35] was used for correction of visibility. RH values greater than 40% and less than 99% were converted to the equivalent visibility in dry conditions. The correction formula (1) is expressed as below [35]:

VIS/VIS(dry) = 0.26 + 0.4285 log(100 − RH)
(1)

where RH is in percent and the log is base 10. When RH < 40%, the visibility did not need to be corrected. If there were natural events such as precipitation or fog at all visibility observation times in a day, the visibility of that day was excluded from the visibility series.

#### 2.2.8. Definition of Haze

A haze day is defined by the following conditions: (1) visibility at 14:00 was less than 10 km; (2) visibility measurements were screened for natural events such as precipitation, dust, fog, mist, and gale using the present weather code; and (3) RH at 14:00 was less than 90% [36]. In line with the visibility analysis, the haze change was analyzed for the period 1980–2011.

#### 2.2.9. Definition of Hot Day

Based on work by Della-Marta *et al.* [37], the threshold of extreme temperature is defined as the long-term daily 95th percentile of daily maximum temperature (*TX*_95_). For each day of the 92 days in the period June to August, a 95th percentile is calculated from a sample of 15 days (7 days either side of the day) using data over the 1961 to 1990 period. This gives a sample of 450 days from which a 95th percentile is calculated. A hot day is defined as a day where the daily summer maximum temperature exceeds the 95th percentile. An index of extreme heat intensity was defined by calculating the total degrees-days of exceedance (DD) [38], obtained using the following expressions formula (2):

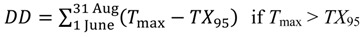
(2)


## 3. Results

### 3.1. Urban Expansion in Zhejiang Province during 1992–2010

**Figure 3:**
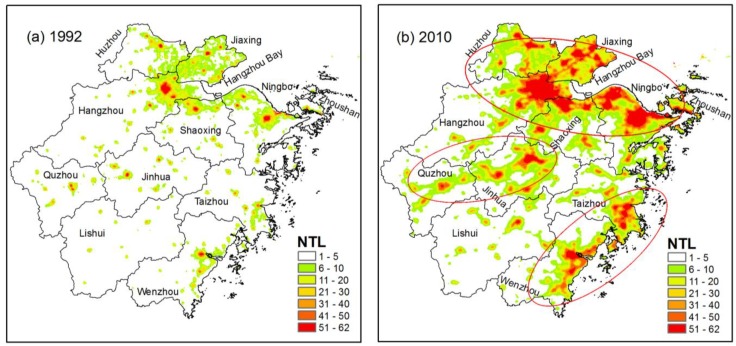
Extent of urban areas in ZJP from (**a**) 1992 to (**b**) 2010 based on inter-calibrated DMSP/OLS nighttime light. Three ellipses indicate three urban clusters (around Hangzhou Bay, the coastal areas of Wenzhou-Taizhou, and the Jinhua-Quzhou Basin).

ZJP has been undergoing a period of rapid urbanization since the late 1970s. The DMSP/OLS NTL data reflect the spatial expansion of urban areas from 1992 to 2010. During the past two decades, central cities, such as Hangzhou, Ningbo, and Wenzhou, have dominated the process of urban expansion in ZJP (Figure 3a) and urban growth has followed the law of “small scale agglomeration, large scale dispersion” [39]. 

**Figure 4:**
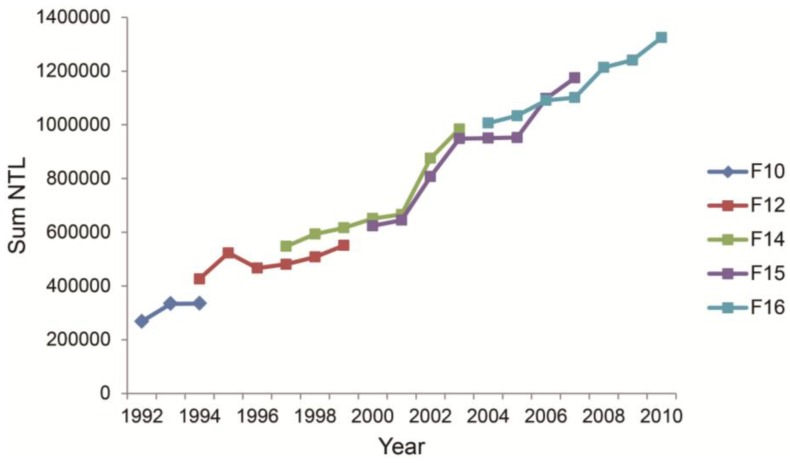
Inter-calibrated sum of DMSP/OLS nighttime lights of ZJP during 1992–2010 from different satellites. Each satellite is designated with a flight number, such as F10 for DMSP satellite number 10.

In 2010, three urban agglomerations initially formed, namely, around Hangzhou Bay, the Wenzhou-Taizhou coastal zone, and the Jinhua-Quzhou Basin (Figure 3b). According to previous studies by Zhao *et al.* [33], a NTL DN threshold value of 10 was chosen for delimiting urban areas in ZJP. Based on the continuous observation of DMSP/OLS, the number of pixels with DN values larger than 10 increases from 10,596 to 22,653 from 1992 to 2000, and then increased to 40,846 in 2010. At the same time, the sum of all NTL DN values are 0.27, 0.67, and 1.32 million for 1992, 2000, and 2010, respectively, indicating that the urbanization process intensified after 2000 (Figure 4), which is in line with the rapid economic development after 2000 shown in Figure 2.

**Figure 5:**
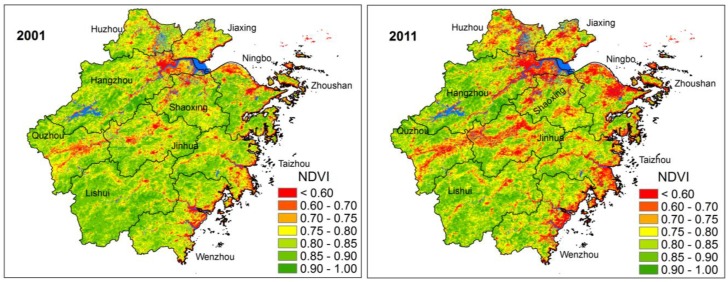
Annual maximum NDVI for 2001 and 2011.

Urban growth has great impacts on vegetation coverage in ZJP. Figure 5 shows the annual maximum NDVI of 2001 and 2011. The decrease of NDVI mainly occurred around the urban areas and towns in the three urban clusters which is consistent with the increase of NTL in Figure 2. The number of pixels with annual maximum NDVI less than 0.6 accounted for 4.2% in 2001 and increased to 8.2% in 2011.

### 3.2. Urbanization Impacts on Extreme Heat Events

**Figure 6:**
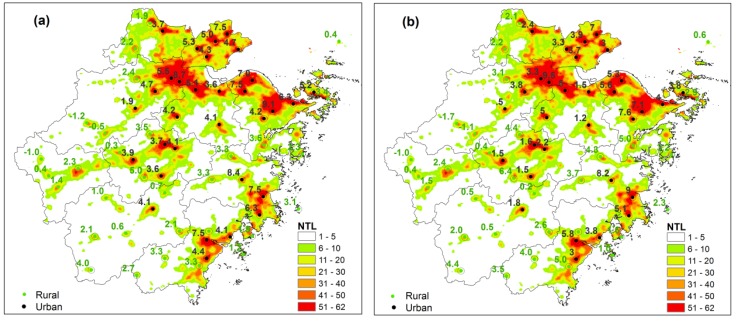
DMSP/OLS nighttime light in 2010 and spatial pattern of (**a**) hot days (day/10 a) and (**b**) hot degree days (°C·day /10 a) trends based on the daily 95th percentile during 1971–2011 in ZJP. Circles indicate significant trends at the 5% significance level.

Extreme heat events have increased in both frequency and intensity during the past four decades under global warming. During 1971–2011 the mean hot days for the 31 rural stations have increased by 2.0 day/10 a and the mean extreme heat intensity (hot degree days) has increased by 2.3 °C·day/10 a. However, both the hot days (Figure 6a) and the extreme heat intensity (Figure 6b) show that the UHI effect caused additional hot days and heat stress in urban stations compared to rural stations. The mean hot days and extreme heat intensity for urban stations have increased by 5.3 day/10 a and 4.2 °C·day/10 a, respectively. The most significant increase occurred in the highly urbanized areas with high NTL DN values, including the city belt around Hangzhou bay, and the Taizhou–Wenzhou and Jinghua-Yiwu city belts. The UHI effect resulting from rapid urbanization in ZJP has significantly increased the duration and magnitude of heat stress within cities. With global warming forecasted to continue into the foreseeable future, the potential exposure of urban residents to extreme heat events will be further enhanced by local factors (especially the UHI) with the development of urbanization.

**Figure 7:**
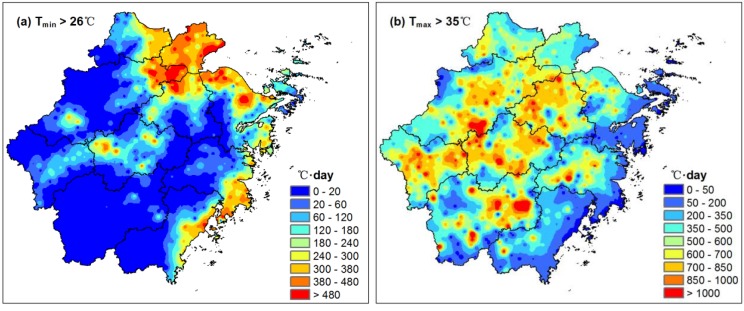
Accumulated temperature with (**a**) daily minimum temperature above 26 °C (**b**) daily maximum temperature above 35 °C during summer (June–August) in ZJP.

Observational data from the dense AWS network were then used to analyze the relationship between summer extreme heat intensity and urbanization level at a regional scale. Figure 7a shows the accumulated daily minimum temperature above 26 °C obtained from the dense AWS network during summer (average for 2008–2012) in ZJP. There are three distinct warming centers in highly urbanized areas, with the most intense values around Hangzhou Bay, the second-highest values in the coastal areas of Wenzhou-Taizhou, and the lowest values in the Jinhua-Quzhou Basin. To the best of our knowledge, this is the first assessment of the nighttime UHI effect during extreme heat events at a regional scale using such a dense AWS network over China. Comparison with the NTL data in Figure 3b shows that the brighter the NTL, the larger the nocturnal heat stress during extreme heat events. The spatial correlation coefficient between the NTL image and the sum of minimum temperature above 26 °C, calculated by ArcGIS software, reaches 0.72. Previous studies suggest the UHI is most pronounced at night and under synoptic high pressure systems with calm and clear conditions [40,41]. Therefore, large cities do not cool off at night during heat waves like rural areas do because of the UHI effect [42], and inhabitants of urban areas may experience sustained heat stresses both day and night during heat waves [43]. Recent satellite monitoring of the heat wave in Paris in 2003 suggested that the highest mortality ratios matched the spatial distribution of the highest nighttime land surface temperature, but were not related to the highest daytime LSTs [44] and supported the influence of night temperatures on the health impact of heat waves in urban areas [45]. Our analysis based on a dense AWS network supports the results from the aforementioned studies that higher nighttime temperatures occur during extreme heat events in urbanized areas in ZJP and inhabitants in urban areas may experience sustained heat stress both day and night during extreme heat events. Recent modeling study by Chen *et al.* [46] during a long-lasting heat wave in Hangzhou, the capital of ZJP, indicated that urban areas have higher near-surface air temperatures during extreme heat events, specifically during the nighttime. Therefore, high-temperature forecasts for large cities must consider the occurrence of abnormal UHI conditions.

In the daytime, the maximum temperature was strongly controlled by latitude, altitude, and distance to the ocean. Therefore, there are no obvious signals of the UHI impact on daytime heat stress indicated by the accumulated daily maximum temperature above 35 °C during summer (Figure 7b) and the spatial correlation coefficient between the NTL and the sum of maximum temperature above 35 °C is only 0.06.

### 3.3. Urbanization and Degraded Visibility and Air Quality

**Figure 8:**
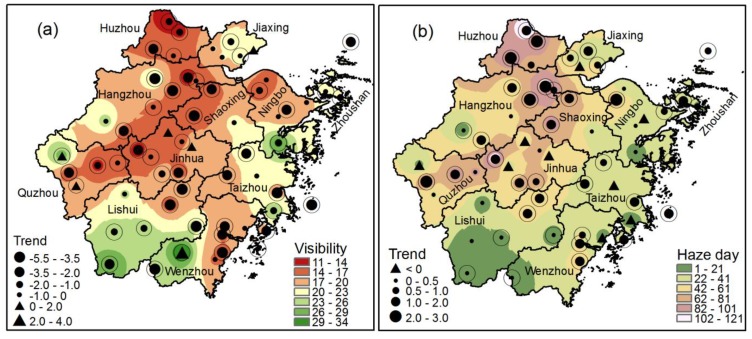
Spatial distribution of (**a**) annual mean visibility (11-year average for the period 2001–2011) and the trend in annual mean visibility (1980–2011); (**b**) annual mean haze days (11-year average for the period 2001–2011) and the trend in annual haze day change (1980–2011).

**Figure 9:**
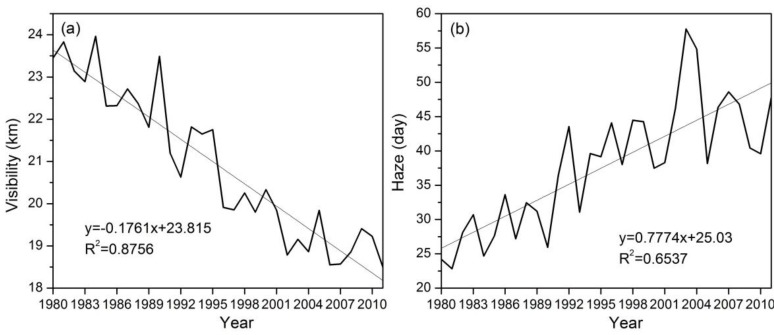
Change in (**a**) average visibility and (**b**) average haze days in ZJP during 1980–2011.

Figure 8a shows the spatial distribution of annual mean visibility in ZJP. Low visibility was observed in urbanized areas in the Hangzhou-Huzhou plain, the Jinhua-Quzhou Basin, and Wenzhou. In addition, degraded visibility occurred at most meteorological stations in ZJP during 1980–2011. Huang *et al.* [19] suggested that visibility can be used as a surrogate for air quality, and decreased visibility was significantly associated with elevated death rates in Shanghai. Therefore, the degraded visibility in urbanized areas in ZJP implies poor air quality and increased health risks for inhabitants of urban areas. In line with the degraded visibility in urbanized areas, more haze days were observed in Hangzhou, Huzhou, and the Jinhua-Quzhou Basin, and annual haze days have increased in most stations, especially in highly urbanized areas (Figure 8b). The average visibility for ZJP has decreased by 1.8 km/10 a and the annual mean haze days have increased by about 7.8 days/10 a during 1980–2011 (Figure 9).

**Figure 10:**
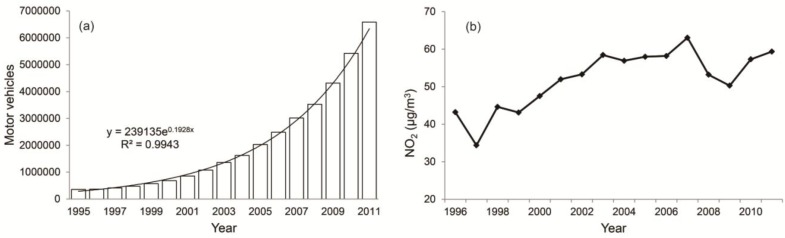
(**a**) Change in the total number of motor vehicles during 1995–2011 in Zhejiang Province and (**b**) change in NO_2_ concentration in Hangzhou City between 1996 and 2011.

Motor vehicle emissions are one of the major sources of air pollution in urbanized areas. The total number of motor vehicles in ZJP showed exponential growth from 1995 to 2011(Figure 10a). By the end of 2011, there were 6.58 million vehicles registered in ZJP, compared to 0.36 million in 1995, an increase of almost 6.22 million vehicles or 1828%. The ever-increasing number of vehicles has become an important contributor to the increasing air pollution. Taking Hangzhou as an example, there were 91,616 motor vehicles in the downtown in 2000, which increased to 821,376 in 2011. Therefore, the increased NO_2_ concentration between 1996 and 2011 in Hangzhou city was mainly attributed to the huge increase in the number of vehicles (Figure 10b). This result is consistent with a previous study in Shanghai [21].

## 4. Discussion and Conclusions

Since China’s economic reforms in the late 1970s, ZJP has experienced rapid urban expansion. This phenomenon has been responsible, to a large extent, for various environmental problems, not only at the city scale but also at the regional scale. In the present study, we focused on the major environmental problems related to human health caused by rapid urbanization in ZJP, namely, exacerbation of extreme heat events, degraded visibility, and air pollution.

During the past two decades, rapid urbanization has occurred in the areas around Hangzhou Bay, the Wenzhou-Taizhou coastal zone, and the Jinhua-Quzhou Basin in ZJP. Along with the dramatic urban growth, a substantial UHI effect during summertime was found in ZJP. The nocturnal UHI effect significantly contributes to the nocturnal heat stress during extreme heat events in the three urban agglomerations. During the daytime, the UHI effect is not a dominant factor for extreme heat intensity. Built-up areas absorb heat during the day and progressively raise minimum nocturnal temperature, which intensifies nighttime heat stress during the extreme heat events. There is a high spatial correlation coefficient at a regional scale between nighttime heat stress and urbanization level with a value of 0.72. Taking a long-term view, the UHI effect caused additional hot days and heat stress intensity in urban stations compared to rural stations over the past four decades in ZJP. As global warming is predicted to continue into the foreseeable future, extreme heat events are very likely to increase in both frequency and intensity. The combined effect of global warming and urbanization will potentially increase the magnitude and duration of extreme heat events within cities. Therefore, the UHI effect should be determined and incorporated in preparing high temperature forecasts in urban areas.

Urbanization also places a heavy burden on local and regional air quality in ZJP. Low visibility can be observed in the three urban clusters, and during the past three decades, degraded visibility was observed at most stations, especially in highly urbanized areas. Meanwhile, more haze days were observed in Hangzhou, Huzhou, and the Jinhua-Quzhou Basin, and the number of annual haze days has increased at most stations, especially in the three urban clusters. During the past three decades, the average visibility for ZJP degraded by 1.8 km/10 a and the mean annual haze days increased by 7.8 days**/**10 a. Furthermore, the NO_2_ concentration in Hangzhou has increased substantially since 1996, which can be mainly attributed to the rapid increase in motor vehicle emissions in urbanized areas. A previous study indicated that more than three-quarters of the urban population is exposed to air quality that does not meet the national ambient air quality standards of China [8]. Therefore, air pollution problems in mega cities and their immediate vicinities will continue to be one of the top environmental concerns in the next decade in China [47].

In summary, ZJP, as a representative of regional rapid urbanization in China, is facing many environmental challenges as its urban growth rate continues to accelerate. Urban residents have experienced an overall decline in quality of life. Managing the tradeoffs between urbanization and environmental protection will be a major challenge for local policy makers. There is an urgent need to incorporate environmental issues into planning China’s urban areas, to reduce the risks of further environmental degradation.
